# Chronic Brucellosis Patients Retain Low Frequency of CD4+ T-Lymphocytes Expressing CD25 and CD28 after* Escherichia* *coli* LPS Stimulation of PHA-Cultured PBMCs

**DOI:** 10.1155/2008/327346

**Published:** 2009-01-25

**Authors:** Panagiotis Skendros, Alexandros Sarantopoulos, Konstantinos Tselios, Panagiota Boura

**Affiliations:** Clinical Immunology Unit, 2nd Department of Internal Medicine, Hippokration General Hospital, Aristotle University of Thessaloniki, Konstantinoupoleos Street 49, 546 42 Thessaloniki, Greece

## Abstract

Chronic brucellosis patients display a defective Th1 response to PHA. We have previously shown that heat-killed B. abortus (HKBA) can downregulate the PHA-induced increase of CD4+/CD25+ and CD14+/CD80+ cells of brucellosis patients. In the present study, we investigate the effect of *E. coli* LPS, as a potent stimulant of monocytes and autologous T-lymphocytes, on the PHA-cultured PBMCs of the same groups of patients. Thirteen acute brucellosis (AB) patients, 22 chronic brucellosis (CB) patients, 11 “cured” subjects, and 15 healthy volunteers were studied. The percentage of CD4+/CD25+ and CD4+/CD28+ T-lymphocytes as well as CD14+/CD80+ monocytes were analyzed by flow cytometry after PBMCs culture with PHA plus *E. coli* LPS. A significant decrease in the percentage of CD4+/CD25+ and CD4+/CD28+ T-lymphocytes was observed in CB compared to AB. In HKBA cultures, compared to *E. coli* LPS-cultures, there was a significant reduction of CD4+/CD25+ T-lymphocytes in all groups and CD14+/CD80+ in patients groups. We suggest that Brucella can modulate host immune response, leading to T-cell anergy and chronic infection.

## 1. INTRODUCTION

Brucellosis is the most common zoonotic disease with worldwide distribution. More than 500 000 new cases are reported annually [1, 2]. It is caused by intracellular pathogens of the genus Brucella that have their natural reservoir in domestic and wild animals [1]. The disease is transmitted to humans by consumption of contaminated dairy products or by occupational contact with infected animals. In addition, Brucella species are thought to be a biowarfare agent and recently reported to be a significant cause of travel and immigration-related infection [1, 3–5].

In Greece, as in other Mediterranean countries, brucellosis remains a major disease of economic and human health importance despite efforts for animal control and eradication projects [2, 4]. Besides minimal brucellosis mortality in humans, the disease causes high clinical morbidity, protean clinical manifestations, and complications, as any organ can be affected [1, 6, 7]. Despite early diagnosis and treatment, approximately 10–30% of patients develop chronic persistence disease which is characterized by atypical clinical picture, chronic fatigue syndrome, and relapses [4, 6–8].

Host protection against Brucella spp. depends on cell-mediated immunity, involving mainly activated macrophages, dendritic cells, and T-cells [9–13]. T-helper 1 (Th1) immune response is essential for the clearance of Brucella infection and Brucella antigens induce the production of Th1 cytokines in humans [9–11, 14]. On the other hand, Brucella can survive and replicate in host macrophages and dendritic cells subverting innate immunity and evading adaptive immune mechanisms [8, 12, 13, 15, 16]. Recent data report that human dendritic cells infected by living Brucellae are poor inducers of human naïve T-lymphocyte proliferation and present low production of interleukin (IL)-12 which is essential to drive a Th1 immune response [13, 16]. Diminished production of Th1 cytokines (IFN*γ* and IL-2) has been associated with T-cell unresponsiveness (anergy) to Brucella antigens and disease chronicity [17–19]. In addition, proliferation response of CD4+ T-lymphocytes to phytohaemagglutinin (PHA) in chronic brucellosis patients was found to be significantly low [20]. Recently, we have shown low frequency of ex vivo and PHA-cultured CD4+ T-lymphocytes expressing IL-2 receptor alpha (CD25) in chronic brucellosis patients [21].

CD80/CD28 costimulation enhances the interaction of antigen/major histocompatibility complex (MHC) with T-cell receptor (TCR) and is critical for an adequate induction and maintenance of the Th1 response [22]. When we studied CD80/CD28 costimulation in human brucellosis, it was indicated that heat-killed B. abortus (HKBA) significantly downregulated the PHA-induced increase of CD80+ monocytes [23]. This effect could be attributed to Brucella lipopolysaccharide (LPS) which is thought to be one of the virulence factors that endow the pathogen with the ability to escape the immune [8, 15, 24]. Brucella smooth LPS is considered to be very important for bacterium survival and replication in the host [24–26]. It is an unconventional, nonendotoxic LPS, as compared with classical LPS from enterobacteria such as *E. coli* [27]. Enterobacteria LPS is a potent stimulant of monocytes for the production of proinflammatory cytokines (such as tumor necrosis factor (TNF)*α*, IL-1*β*) and the expression of CD80 [28]. Moreover, it induces the IFN*γ* secretion, as well as, the upregulation of CD25 and CD28 by autologous T-lymphocytes [29–32].

Thus, we decided to investigate the influence of *E. coli* LPS on CD25 expression and CD80/CD28 costimulation of PHA-stimulated peripheral blood mononuclear cells (PBMCs) from brucellosis patients and compare the findings with our previous results regarding the HKBA stimulation of PBMCs in the same groups of patients [23].

## 2. MATERIALS AND METHODS

### 2.1. Subjects

Sixty one unrelated subjects were included in the study: (a) 35 brucellosis patients; (b) 11 subjects who had a previous history of brucellosis and were cured at least 2 years ago; (c) 15 healthy age- and gender-matched volunteers who were used as controls. Patients were divided in acute (AB) and chronic (CB) brucellosis groups according to disease history, clinical picture, and laboratory findings [21, 23].

The AB group included 13 consecutive patients (Table 1). All AB patients had a disease duration ≤8 weeks (mean ± SD, 3.9 ± 2.6 weeks). The diagnosis was based on a compatible clinical picture (Table 2) in combination with high serum titres of antibrucellar antibodies or a fourfold increase of the initial titres in two-paired samples drawn 2 weeks apart. In addition, in 6 out of 13 AB patients, Brucella melitensis was isolated in blood culture. In 2 patients, brucellar DNA was detected by PCR analysis in the blood and the serum.

The CB group comprised 22 patients (Table 1). All CB patients had a disease duration ≥12 months. Twelve patients were suffering from the relapsing type of chronic disease (CB1 subgroup, mean disease duration 36.3 ± 18.8 months) which is characterized by periodic attacks of fever, chills, and arthalgias/myalgias [6, 7, 21, 23] (mean number of relapses 2.3 ± 0.7). At the time of study entry, CB1 patients were under relapse in accordance to the clinical picture and the high titres of antibrucellar antibodies. Nine of 12 CB1 patients had also positive PCR analysis in the blood. The variable type of the chronic disease was presented by 10 of the 22 chronic brucellosis patients (CB2 subgroup, mean disease duration 32.4 ± 15.9 months). CB2 subgroup patients experienced no relapses but displayed atypical symptoms (Table 2) and persistently high serum antibrucellar antibody titres [6, 7, 21, 23].

High antibrucellar antibody titres were considered for the Wright agglutination test ≥1 : 320, for the Coombs agglutination test ≥1 : 320, and for the complement fixation test ≥1 : 32.

“Cured” from brucellosis subjects (Table 1) remained symptomless and with negative antibrucellar antibodies for at least 2 years. Healthy volunteers (Table 1) were tested serologically for brucellosis and found to be negative.

All CB patients and “cured” subjects had received initial antibiotic treatment including mainly streptomycin 1gr/day combined with rifampicin 600 mg/day plus doxycycline 200 mg/day for 2 weeks and rifampicin 600 mg/day plus doxycycline 200 mg/day for 6 to 10 weeks thereafter. In 8 of 22 CB patients and 3 of 11 “cured” individuals, streptomycin was not administered. In a CB1 patient who suffered from spondylitis (Table 2) treatment lasted for 6 months. In addition, most of CB patients had received more than one antibiotic course and alternative therapeutic approaches were used such as cotrimoxazole or ciprofloxacin in combination with doxycycline and/or rifampicin. Patients with inadequate treatment were not included in the study.

The exclusion criteria of the study were

- coexistence of other infectious, neoplasmatic, or autoimmune disease;
- administration of antibiotic or immunostimulating agents for at least 30 days before entering the study;
- recent (≤6 months) vaccination;
- pregnancy.

Informed consent was obtained from all subjects enrolled in the study.

### 2.2. Cell cultures

Twenty mL heparinized venous blood was collected in sterile tubes and PBMCs were isolated using density gradient centrifugation (Histopaque-1007, Sigma Laboratories, St. Louis, Mo, USA). The viability of PBMCs was determined to be greater than 95%, as indicated by Trypan blue dye exclusion (Sigma Laboratories, St Louis, Mo, USA).

We used LPS from *E. coli* serotype 0111 : B4 (Sigma Laboratories, St. Louis, Mo, USA). This serotype does not share high antigenic similarity with brucella species [33].

Preliminary experiments in PBMCs from healthy volunteers were carried out to standardize the time and dose of PHA and *E. coli* LPS needed for the optimal expression of CD25, CD80, and CD28. In accordance with previous reports, PBMCs were stimulated with 1, 3, and 10 *μ*g *E. coli* LPS per mL of culture medium [34–36] and no differences were noticed in the expression of the CD80 molecule. Finally, we decided to use the 3 *μ*g/mL concentration, as it has already been used in another brucellosis study on human monocytes [34].

PBMCs were cultured in triplicate in the presence of 5 *μ*g/mL PHA plus 3 *μ*g/mL *E. coli* LPS in 24-well plates (Costar, Boston, Mass, USA). PBMCs were 1 × 10^6^ cells per well of culture plate. Each well contained 1 mL of culture suspension. Culture medium consisted of RPMI-1640 (Gibco Laboratories, Paisley, UK) supplemented with 10% fetal calf serum (Gibco Laboratories, Paisley, UK), 2 mmol/l l-glutamine (Sigma Laboratories, St. Louis, Mo, USA), 100 IU/mL penicillin (Sigma Laboratories, St. Louis, Mo, USA), and 100 mg/mL streptomycin (Sigma Laboratories, St. Louis, Mo, USA). The cultures were kept at 37°C in a humidified 5% CO_2_ atmosphere for 48 hours. Approximately 3 × 10^6^ cells per mL of sample were pelleted in a round-bottomed centrifuge tube and washed in RPMI-1640. The pellet was resuspended and 100 mL (~3 × 10^5^ PBMCs) was stained immediately with 20 *μ*L of the appropriate monoclonal antibody (MAB). After 15 minutes incubation at room temperature, the PBMCs were fixed by using Immunoprep/Q-prep protocol (Coulter, Hialeah, Fla, USA).

The dual staining method (synchronous two-color fluorescence analysis) was used. The MABs included fluorescein isothiocyanate (FITC)-conjugated anti-CD4 (clone 13B8.2) and anti-CD80 (clone MAB104) as well as phycoerythrin (PE)-conjugated anti-CD25 (clone B1.49.9), anti-CD28 (clone CD28.2), and anti-CD14 (clone RMO52). Appropriate isotype controls included FITC and PE conjugates of IgG1 (clone 679.1Mc7) and IgG1 (clone 679.1Mc7) mouse MABs which were used to assess the nonspecific binding and to set the threshold between positively and negatively stained cell populations. All MABs were obtained from Immunotech (Marseille, France).

### 2.3. Flow cytometry

The following parameters were analyzed by flow cytometry after PBMCs stimulation with PHA plus E. coli LPS:

- the percentage of CD4/CD25 and CD4/CD28 double-positive T-lymphocytes;
- the percentage of monocytes (CD14) expressing CD80.

PBMCs suspension was used rather than purified monocytes because we intended to investigate the impact of Brucella infection on monocyte–T-cell interaction (costimulation from both sides of cell interaction) [23].

For the flow cytometric analysis, EPICS XL (Coulter Electronics, Hialeah, Fla, USA) was used. Lymphocytes were gated using forward scatter (FS) versus side scatter (SS) dot plots. CD45 FITC staining (clone ALB12, Immunotech, Marseille, France) was used to confirm the purity of the lymphocyte population (>97%). Monocytes were characterized and gated both in an FS versus SS dot plot and in a dot plot with SS versus PE-labeled CD14 antibody (purity >90%). At least 5000 lymphocytes and 2000 monocytes were acquired and analyzed for their fluorescence properties. The percentages of surface antigen-stained positive cells were calculated as the percentage of cells that stained above the fluorescence value obtained by isotype control antibodies. Only CD14 strongly positive monocytes were analyzed with respect to percentage expression of CD80 FITC. Results of the mean fluorescence intensity did not provide any additional information, and are therefore not reported.

### 2.4. Statistical analysis

Parametric statistical tests were applied as the variables were distributed normally (Kolmogorov-Smirnov test). Data were analyzed by Student's *t*-test or paired *t*-test (for paired data) using SPSS software (SPSS Inc., Chicago, Ill, USA) and were represented as mean ± SD. *P*-values < .05 were considered to be statistically significant.

## 3. RESULTS

There was a significant increase in the percentage of CD4+/CD25+ T-lymphocytes in AB patients compared to controls (*P* = .027). On the other hand, a significant decrease in the percentage of CD4+/CD25+ T-lymphocytes was observed in CB group of patients compared to AB (*P* = .006) (Table 3(a)).

Concerning CD4+/CD28+ T-lymphocytes, no differences were found between AB patients and “cured” subjects or controls. Similarly, CD4+/CD28+ T-lymphocytes in CB patients did not significantly differ in comparison to nonpatients groups. When CB group of patients compared to AB, a significant decrease in the percentage of CD4+/CD28+ T-lymphocytes of CB was observed (*P* = .024) (Table 3(a)).

T-lymphocytes subsets did not significantly differ between the CB1 and CB2 subgroups, however the diminished percentage of CD4+/CD25+ T-lymphocytes in CB patients was related to the CB1 subgroup (*P* = .008). In the same manner, CB1 patients showed significantly lower frequency of CD4+/CD28+ T-lymphocytes (*P* = .039) (Table 3(a)).

Regarding the frequency of CD14+ monocytes expressing CD80 costimulation molecule, a significant increase was found in both of brucellosis groups in comparison to “cured” (*P* = .002 and .001, resp.) and controls (*P* < .001).

No differences of the percentage of CD80+ monocytes were observed between the CB1 and CB2 subgroups. However, there was a significant increase in the percentage of this cellular population between both subgroups of CB patients and controls (*P* = .003 and .004, resp.) (Table 3(a)).

**Figure 1:**
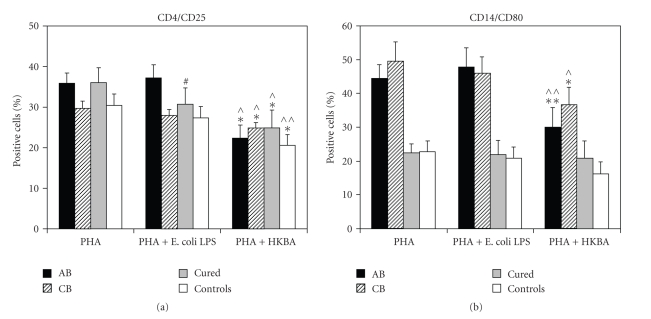
Comparison of the PHA-cultures. HKBA addition downregulates (a) the percentages of CD4+/CD25+ T-cells in all groups and (b) CD14+/CD80+ monocytes in brucellosis patients. Data represent mean values+SD (SE). *^*#*^P* ≤ .05; *PHA versus PHA + E. coli LPS*, ^⋀^ *P* ≤ .05; ^⋀⋀^ *P* < .001; *PHA versus PHA + HKBA,* *P ≤ .05; **P < .001; *PHA + E. coli LPS versus PHA + HKBA*.

We compared the above results with the findings yielded from our previous studies (on the same cultured PBMCs stimulated simultaneously with PHA alone or PHA plus HKBA, Figure 1) [21, 23]. The results between PHA alone and PHA plus *E. coli* LPS-cultured PBMCs were similar. On the contrary, when HKBA-cultures, compared to *E. coli* LPS-cultures, there was a significant reduction of CD4+/CD25+ T-lymphocytes in all groups (AB *P* = .005, CB *P* = .042, “cured” *P* = .029, controls *P* = .007) and CD14+/CD80+ monocytes in patients groups (AB *P* < .001, CB *P* = .05) (Figure 1).

## 4. DISCUSSION

Brucella acquires the ability to establish chronic infection and has evolved strategies to actively modulate the host immune response [8, 10, 13, 15, 16]. Chronic brucellosis patients display a defective Th1 response to PHA characterized by low-proliferation response of CD4+ T-lymphocytes, diminished production of IL-2 and IFN*γ*, and low frequency of CD4+/CD25+ T-cells compared to acute brucellosis patients [17–21]. In this study, we investigated the effect of a potent monocytic stimulant (*E. coli* LPS) on the PHA-cultured PBMCs of patients with different clinical forms of brucellosis, especially focusing on chronic disease.

*E. coli* LPS stimulation of PHA-cultured PBMCs yielded a significant increase in the percentage of CD4+/CD25+ T-lymphocytes in AB patients as compared to controls. This finding comes in agreement with previous data suggesting increased levels of soluble CD25 and upregulation of CD4+/CD25+ T-cells in acutely ill brucellosis patients [21, 37, 38]. Generally, previous in vitro and in vivo human studies showed that LPS stimulation of normal T-lymphocytes upregulates the CD25 expression and the secretion of IL-2 and IFN*γ* by them. Thus, LPS is thought to be a potent inducer of human T-cell proliferation and Th1 cytokine production [29–32]. However, increased percentage of CD4+/CD25+ cells in AB compared to CB is rather LPS independent, since, as we have shown previously, PHA-alone cultures displayed similar result [21] (Figure 1). The higher percentage of CD4+CD25+ T-cells in acute brucellosis could be attributed to the abundant milieu of proinflammatory cytokines observed in the acute disease [37, 39, 40].

On the other hand, the low frequency of CD4+/CD25+ T-lymphocytes in CB compared to AB group could be explained as an effect of chronic disease. Chronic brucella infection could lead to a defective in vitro blastogenesis of CD4+ T-lymphocytes due to low-proliferation response, reduced production of IFN*γ* and IL-2, and switching toward Th2 response characterized by an increased percentage of CD3+/IL-13+ T-lymphocytes [17–20]. It had been suggested that IL-13 can downregulate macrophage functions, such as the production of IL-12 and the expression of inducible nitric oxide synthase in response to LPS [19, 41].

The reduction of CD4+/CD25+ T-cells percentage after PHA plus *E. coli* cultures was more pronounced in chronic relapsing brucellosis patients (CB1 subgroup), suggesting that relapses in brucellosis might be associated with disturbances of IL-2/IL-2 receptor system [21].

Stimulation of human T-lymphocytes by LPS was found to be MHC-unrestricted, but strongly dependent on costimulatory signals provided by CD80/CD28 interactions [42]. CD80 seems to be crucial for the activation of T-cells by monocytes, since monocytes expressing CD86, but not CD80 after LPS stimulation, were unable to stimulate human T-cells [42]. Moreover, endotoxin administration to healthy subjects increases T-lymphocytes CD28 expression [32].

Noteworthy, when PHA-cultured PBMCs were stimulated by *E. coli* LPS, CB patients and especially CB1 subgroup (relapsing patients) displayed significantly lower percentage of helper T-lymphocytes expressing CD28 costimulation receptor in comparison to acutely ill patients, ranging at the levels of controls. In our previous study [23], HKBA stimulation of PHA-cultured PBMCs did not increase the frequency of CD4+/CD28+ T-cells in chronic relapsing brucellosis (Table 3(b)).

It could be expected that a potent pathogen-associated molecule pattern (PAMP) stimulation, either by *E. coli* LPS or HKBA, should lead to the upregulation of CD28+ T-cells in CB1 patients. It seems that chronic relapsing brucellosis patients are unresponsive/anergic to nonspecific or brucellar antigens as it was shown previously [17, 19, 20, 43, 44]. On the other hand, the effect of *E. coli* on PHA-cultured CD4+/CD28+ T-cells could be attributed to “LPS tolerance phenomenon”, where prior sublethal exposure to LPS results in a state of tolerance to further LPS challenge [45]. LPS tolerance is a state of altered immunity characterized by decreased production of macrophage-derived cytokines, such as TNF*α*, IL-1*β*, and IL-6, as well as lymphocyte-derived IFN*γ* [46, 47]. As a result LPS tolerant, macrophages have a markedly impaired ability to induce IFN*γ* secretion by autologous T-cells and NK-cells [48]. It could be assumed that in chronic relapsing brucellosis patients prior exposures to Brucella LPS, as a result of relapses during the chronic stage, leads to “cross tolerance” (low percentage of CD4+/CD28+ T-cells) to further in vitro *E. coli* LPS challenge. Nevertheless, it is not known if chronic brucellosis patients present high incidence of gram negative enterobacteria infections.

*E. coli* addition in PHA cultures did not further enhance the increase in the percentage of CD14+/CD80+ monocytes in both groups of brucellosis patients. The percentage of CD14+/CD80+ cells with PHA plus LPS in PBMCs cultures were similar to that obtained with PHA alone [23] (Figure 1). It could be speculated that patients monocytes display maximal activation when exposed to PHA in cultures and cannot be further activated by *E. coli* LPS addition. The inflammatory microenvironment of the infection may also contribute to this effect [49, 50]. Specifically, in chronic brucellosis patients, high percentage of CD14+/CD80+ monocytes in PHA and PHA plus *E. coli* LPS cultures might compensate for an ineffectual CD4+ T-cell response characterizing these form of disease [17–21].

Previously, we stimulated PHA-cultured PBMCs from brucellosis patients with HKBA in order to investigate any possible effect of brucellar antigen/s (mainly LPS) on the expression of CD25 and CD80/CD28 costimulation molecules [23]. We used whole cells of HKBA in PHA cultures in order to evaluate the immunomodulative potential of the total brucellar antigenic structure. HKBA addition in comparison to *E. coli* LPS stimulation led to a significant reduction of CD4+/CD25+ T-lymphocytes and CD14+/CD80+ monocytes PBMCs in a dose-related manner supporting an immunomodulating effect of brucella on human PBMCs (Figure 1) [23].

Actually, in human macrophages, smooth LPS protects Brucella from phagosome-lysosome fusion, bactericidal cationic peptides, and complement attack and, moreover, is able to induce IL-10 (anti-Th1 cytokine) by PBMCs of healthy individuals [8, 24, 51–55]. Interestingly, recent data suggest that the smooth strains of living Brucella, in contrast to *E. coli*, prevent the maturation of infected human dendritic cells and impair their capacities to present antigen to naïve T-cells and to secrete IL-12 [16, 26]. Moreover, other results suggest that HKBA also inhibits major histocompatibility complex class II expression and antigen processing in human monocytes [56].

*In conclusion*, T-lymphocytes of chronic brucellosis patients retain low percentages of CD4+/CD25+ and CD4+/CD28+ T-lymphocytes after potent stimulation of PHA-cultured PBMCs by *E. coli* LPS, although the frequency of CD14+/CD80+ monocytes remains increased. Based on the findings revealed after the comparison of the PBMCs PHA-cultures treated with specific (HKBA) or no specific (*E. coli* LPS) antigen, we suggest that Brucella modulates both functional arms (innate and adaptive) of human immune system, leading to T-cell anergy and chronic infection.

## Figures and Tables

**Table 1:** Demographic data of the groups (AB, CB, “cured,” controls) and CB subgroups (CB1, CB2) studied.

	AB	CB	CB1	CB2	Cured	Controls
*n*	13	22	12	10	11	15
Female	5	2	1	1	4	4
Male	8	20	11	9	7	11
Age (mean ± SD, years)	44.7 ± 21.4	48.1 ± 15.6	45.3 ± 14.8	51.6 ± 16.6	50.3 ± 16.7	43.8 ± 16.3

**Table 2:** Clinical characteristics of the patients studied.

Symptoms	AB (*n* = 13)	CB1 (*n* = 12)	CB2 (*n* = 10)
Fever	13	3	
Sweating	11	2	1
Chills	9	1	
Malaise/fatigue	8	8	9
Arthalgias	6	5	1
Lumbar pain	4	3	1
Headache	4		
Myalgias	2	3	
Depression			1

Focal disease			

Spondylitis	1	1	
Sacroiliitis		1	
Epididymoorchitis	1		
Meningoencephalitis	1		

**Table 3:** Percentage of CD4+/CD25+, CD4+/CD28+ lymphocytes, and CD80+ cells of CD14+ population, in PHA-cultures with (a) *E. coli* LPS and (b) HKBA (results are presented as mean ± SD). *NS: nonsignificant.*

(a)	* E. coli LPS cultures*	*n*	%CD4+/CD25+	%CD4+/CD28+	%CD14+/CD80+
	AB	13	37.2 ± 11.6	43.4 ± 11.3	47.9 ± 19.9
	CB	22	27.9 ± 6.9	35.7 ± 8.0	45.9 ± 23.2
	Cured	11	30.8 ± 12.8	39.8 ± 17.2	21.8 ± 14.4
	Controls	15	27.4 ± 10.5	36.5 ± 14.9	20.9 ± 12.5

			*P (AB versus cured) = NS*	*P (AB versus cured) = NS*	*P(AB versus cured) = .002*
			*P (AB versus controls) = .027*	*P (AB versus controls) = NS*	*P (AB versus controls) < .001*
			*P (CB versus cured) = NS*	*P (CB versus cured) = NS*	*P (CB versus cured) = .001*
			*P (CB versus controls) = NS*	*P (CB versus controls) = NS*	*P (CB versus controls) < .001*
			*P (AB versus CB) = .006*	*P (AB versus CB) = .024*	*P (AB versus CB) = NS*

	CB1	12	26.3 ± 5.9	34.5 ± 8.7	49.9 ± 26.5
	CB2	10	29.9 ± 7.9	37.1 ± 7.2	41.1 ± 18.7

			*P (CB1 versus AB) = .008*	*P (CB1 versus AB) = .039*	*P (CB1 versus CB2) = NS*
					*P (CB1 versus controls) = .003*
					*P (CB2 versus controls) = .004*

(b)	*HKBA cultures*				

	AB	13	22.4 ± 11.5	35.9 ± 14.5	30.0 ± 20.8
	CB	22	24.8 ± 6.5	38.8 ± 11.2	36.5 ± 25.5
	Cured	11	24.8 ± 14.8	38.7 ± 17.9	20.9 ± 16.7
	Controls	15	20.6 ± 10.3	35.6 ± 16.9	16.3 ± 13.6

			*NS*	*NS*	*P (AB versus controls) = .047*
					*P (CB versus controls) = .004*

	CB1	12	24.9 ± 7.6	38.3 ± 13.3	46.6 ± 26.6
	CB2	10	24.7 ± 5.1	39.3 ± 8.7	24.4 ± 18.7

			*NS*	*NS*	*P (CB1 versus CB2) = .038*
					*P (CB1 versus cured) = .012*
					*P (CB1 versus controls) = .003*
